# Bandage lens for treatment of corneosclera melting two weeks after pterygium surgery: A case report

**DOI:** 10.1097/MD.0000000000025348

**Published:** 2021-04-09

**Authors:** Tao Gao, Xiaojing Fan, Yaying Wu, Xiuming Jin

**Affiliations:** Eye Center, The Second Affiliated Hospital of Zhejiang University School of Medicine, 88 Jiefang Road, Hangzhou, China.

**Keywords:** bandage lens, corneal melting, pterygium, scleral melting

## Abstract

**Introduction:**

Pterygium is a common chronic ocular surface condition in ophthalmology.

At present, the main treatment modality is surgical resection. Although the recurrence rate can be controlled to varying degrees, some patients can still develop serious complications, such as scleral melting, corneal melting, and even corneal perforation.

We report a case of severe corneal and scleral melting after pterygium surgery treated with a bandage lens.

**Patient information:**

A 60-year-old male who developed corneoscleral melting after pterygium surgery.

**Diagnosis:**

This patient was diagnosed with corneoscleral melting.

**Interventions:**

This patient was treated with a bandage lens and eye drops.

**Outcomes:**

He was treated with a bandage lens, and the tear break-up time (BUT) was prolonged. After 12 days the cornea and sclera were completely cured and the bandage lens was removed after one month.

**Conclusion:**

After pterygium surgery, various factors affect the occurrence of serious complications of autolysis. Mainly on ocular parts, such as the cornea and sclera, a bandage lens can stabilize the ocular surface tear film and prolong the tear break-up time (BUT), effectively prevent corneoscleral melting and promote corneoscleral cure.

## 1 Introduction

Pterygium is a common chronic ocular surface condition in ophthalmology. It is related mainly to abnormal ocular surface function.

At present, the main treatment modality is surgical resection. Currently, there are a variety of available surgical methods, such as limbal stem cell transplantation, amniotic membrane transplantation, conjunctival transplantation, and the intraoperative application of antimetabolites. Although the recurrence rate can be controlled to varying degrees, some patients will develop serious complications, such as scleral melting, corneal melting, and even corneal perforation.

Some scholars have found that corneoscleral melting is associated with simple pterygium excision after combination use of mitomycin C (MMC), exposed scleral surface, excessive cauterization for hemostasis, and systemic connective tissue disease.^[1–3]^

This report is intended to present a patient with severe complications of corneoscleral melting after pterygium surgery. He was already treated with a bandage lens combined with eye drops to control ocular surface inflammation, the tear break-up time (BUT) was prolonged, and the cornea and sclera were successfully repaired.

## 2 Case presentation

A 60-year-old male underwent pterygium excision in a local hospital for two weeks. Postoperative medications included 0.5% levofloxacin eye drops 4/day (5 ml: 24.4 mg Santen Pharmaceutical Co, Ltd), pranoprofen eye drops 4/day (Senju Pharmaceutical Co., Ltd. Fukusaki Plant), and recombinant bovine basic fibroblast growth factor eye gel 4/day (Zhuhai Yisheng Bio-Pharmaceutical Co. LTD). Two weeks after surgery, the patient developed redness and photophobia in the right eye, with an aggravated grinding sensation, and gradually developed blurred vision. He visited our hospital to seek further treatment.

**Figure 1:**
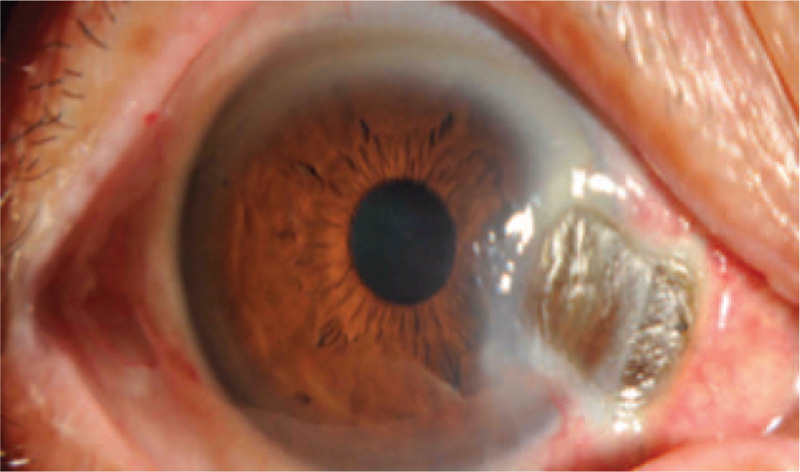
The conjunctiva of the operated eye had mixed hyperemia (+ +), the graft at the corneal limbus of the nasal side was dissolved, the lesion area was dry and depressed, the sclera had become thinner, the uveal coat was transparent, the suture below the nasal side was in place, the grayish white turbidity was visible in the periphery, fluorescein staining (+), no stable tear film was found in the dry pit, there was a stream sign (−) upon gentle pressing.

Signs by slit-lamp examination (Fig. 1): The conjunctiva of the operated eye had mixed hyperemia (+ +), the graft at the corneal limbus of the nasal side was dissolved, the lesion area was dry and depressed, the sclera had become thinner, the uveal coat was transparent, the suture below the nasal side was in place, the grayish white turbidity was visible in the periphery, fluorescein staining (+), no stable tear film was found in the dry pit, there was a stream sign (–) upon gentle pressing, KP (–) was noted, the anterior chamber was clear, and the depth was fair.

The patient reported that he strictly followed the prescribed postoperative medication regimen and denied any other systemic diseases, such as rheumatism or Sjogren syndrome (SS). Corneal and scleral smears from the ulcer lesion were searched for evidence of bacterial, viral, and fungal infections, and the results were negative. Whole blood count, electrolytes, and inflammatory markers were also within the normal range, and immunological tests did not show any pathologically elevated anti-nuclear antibodies (ANAs) or anti-neutrophil cytoplasmic autoantibodies (ANCAs).

The medication regimen of the patient was changed after visiting the hospital. He was treated with infection-prevention therapy comprising 0.3% sodium hyaluronate eye drops 4/day (Santen Pharmaceutical Co, Ltd) and ofloxacin eye ointment 3/day (Santen Pharmaceutical Co, Ltd), and he was asked to wear a corneal bandage lens (Bausch & Lomb PureVision 2 USA) to stabilize the tear film environment on the ocular surface.

**Figure 2:**
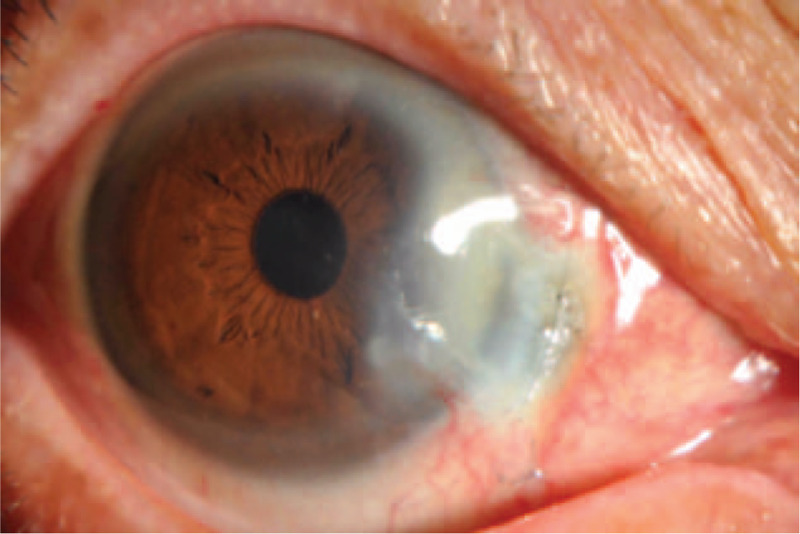
The corneoscleral depressed area was gradually repaired, with grayish white changes; the lesion area was moist; the surrounding bulbar conjunctiva crawled along the sclera of the exposed area; and the pigment membrane was faintly observed.

**Figure 3:**
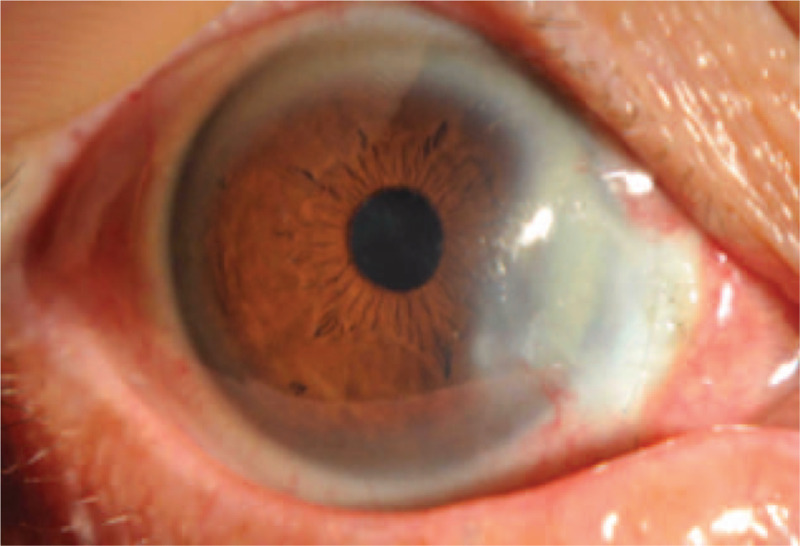
The corneoscleral depressed area was gradually repaired, with grayish white changes; the lesion area was moist; the surrounding bulbar conjunctiva crawled along the sclera of the exposed area; and the pigment membrane was faintly observed.

**Figure 4:**
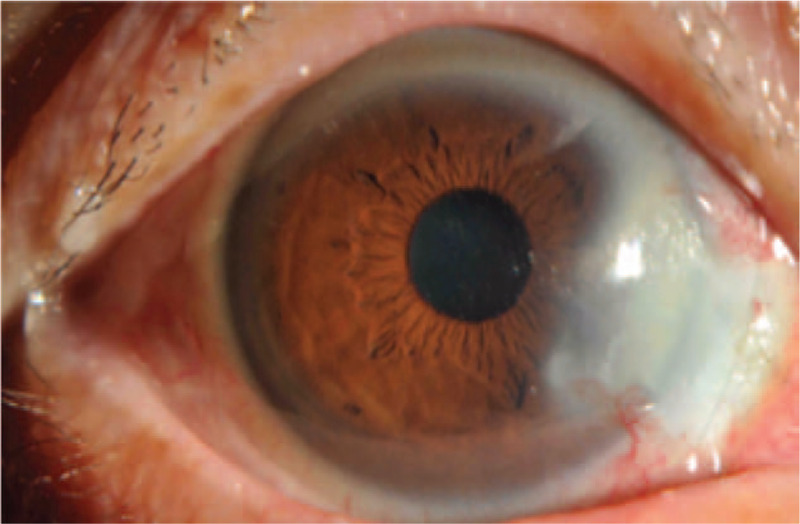
The grayish white lesions in the corneal and scleral defect areas were thickened, and the surface of the lesion was gradually smooth.

**Figure 5:**
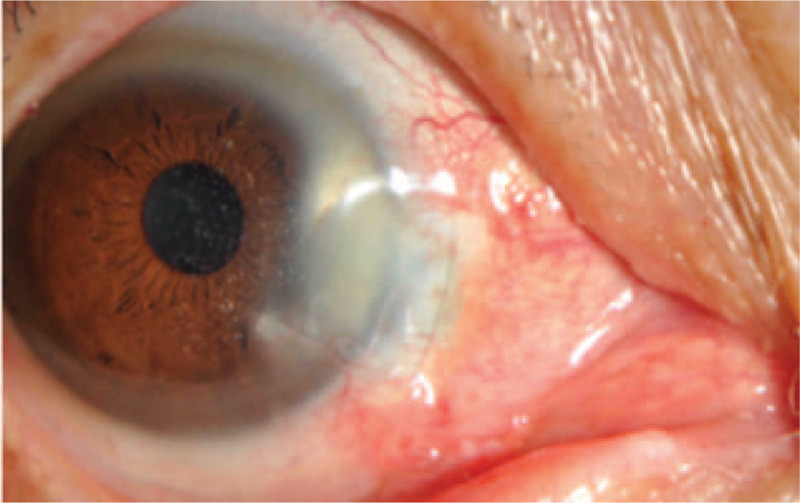
There was increased transparency of the grayish white lesion area, with crawling of a small number of blood vessels in the surroundings.

**Figure 6:**
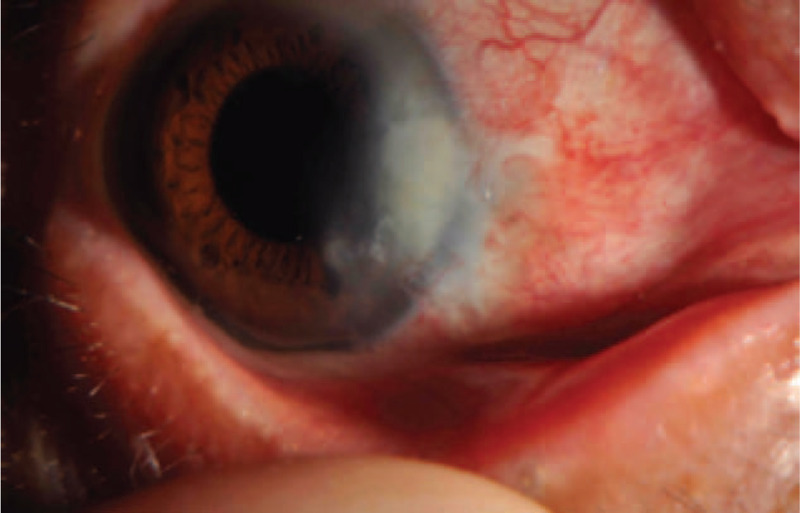
Corneal scars, new blood vessels on the surface of the scleral defect area, and indistinct boundaries.

On the 1st (Fig. 2) and 2nd (Fig. 3) days, reexamination showed that the bandage lens was in place; the corneoscleral depressed area was gradually repaired, with grayish white changes; the lesion area was moist; the surrounding bulbar conjunctiva crawled along the sclera of the exposed area; and the pigment membrane was faintly observed. The tear break-up time (BUT) was 0.5 s. Reexamination on day 5 (Fig. 4) showed that the bandage lens was in place, the grayish white lesions in the corneal and scleral defect areas were thickened, and the surface of the lesion was gradually smooth. The BUT was 1 s. Reexamination on day 12 (Fig. 5) showed that the bandage lens was in place and that there was increased transparency of the grayish white lesion area, with crawling of a small number of blood vessels in the surroundings. The BUT was 2 s. Reexamination after 1 month (Fig. 6) showed that the bandage lens was removed. There were residual corneal scars, new blood vessels on the surface of the scleral defect area, and indistinct boundaries. The BUT was 2 s.

The patient was advised to strictly follow the prescribed postoperative medication regimen. After the use of the bandage lens combined with eye drops, the cornea and sclera were completely cured, the tear break-up time (BUT) was prolonged, and the pterygium did not recur without other ocular complications.

## 3 Discussion and conclusions

This case illustrates the serious complications of corneoscleral melting after pterygium excision. After the use of a bandage lens to stabilize the ocular surface tear film and the combination with eye drops, the tear break-up time (BUT) was prolonged, and the cornea and sclera were completely cured. Recent studies have found that ocular surface traits such as abnormal tear film and insufficient tear secretion have an important association with the occurrence of pterygium.^[4]^ Julio^[5]^ revealed the cause of decreased tear film stability due to pterygium. The formation of pterygium causes an increase in tear osmotic pressure and destroys the integrity of the ocular surface, resulting in reduction of conjunctival goblet cells, in turn cause a change in the normal arc of the ocula and formation of an unsmooth surface, affecting the surface tension of the tear film leading to tear film instability.

Many ocular surgeries can cause tear film instability and damage to the ocular surface due to abnormal quality and quantity of tears or abnormal tear motility and even lead to surgery-induced dry eye. Some scholars have shown this after pterygium excision. There is decrease in tear film secretion, stability,^[6]^ and proneness to a series of corneal irritation symptoms, such as conjunctival congestion, which are generally relieved in a few days. Corneal dry pit spots in a small number of patients are easily ignored by clinicians. However, in severe cases, there may be secondary corneal and scleral infections and even perforation. When the bulbar conjunctiva adjacent to the limbus is edematous or bleeding and, therefore, bulging after surgery, the eyelids cannot contact the corneal area beside the elevation, and no new tear film can be formed in this area at each blink; thus, the tear film is easy to rupture, and the cornea in this area becomes dry and dehydrated, which eventually lead to formation of a dry pit. In this case, the patient was likely to have a corneal dry pit in the early stage because the patient generally had no obvious subjective symptoms, and the clinician did not pay enough attention. Gradually, the lesion developed massive melting and depression of the cornea and sclera.

In this case, after exclusion of bacterial and fungal infections, a corneal bandage lens was worn to restore the integrity of the ocular surface structure and maintain the stability of the ocular surface tear film.

1. This lens was intended to reduce the patient's pain, and studies have shown that the use of a bandage lens in the treatment of ocular surface diseases can effectively relieve the patient's ocular pain and foreign body infection-caused irritation symptoms and can effectively promote the recovery of the patient's corneal epithelium and wound healing.^[7]^
2. A corneal bandage lens, especially a silicone hydrogel corneal bandage lens, is a silicone material with good water absorption capacity that can simulate the lipid layer, help retain water, and reduce tear evaporation, thereby stabilizing the tear film^[8]^ and promoting healing of the lesion area.
3. Corneal bandage lenses have a higher wettability and oxygen permeability, which can better penetrate antibiotics, hormones, artificial tears and other eye drops and will not interfere with the efficacy of eye drops; in addition, they not only reduce the incidence of infection but also play a stable role in the new corneal epithelium and improve the speed of corneal epithelial repair.^[9]^

Therefore, the patient chose to wear a corneal bandage lens to maintain the stability of the ocular surface tear film, in combination with eye drop treatment, to further promote corneoscleral healing.

The patient was successfully treated for serious complications of corneoscleral melting after pterygium surgery by wearing a corneal bandage lenses combined with eye drops. He was satisfied with this kind of treatment. The use of a bandage lens can restore the integrity of the cornea and sclera and maintain the stability of tear film, and its combination with eye drops will not only significantly improve the patient's comfort but also serve as a safe and effective modality of treatment. Clinicians may also consider preventing the formation of a corneal dry pit by asking patients to wear a corneal bandage lens after pterygium surgery, which in turn prevents serious complications of corneoscleral melting.

## Author contributions

**Writing – original draft:** Tao Gao, Xiaojing Fan.

**Writing – review & editing:** Tao Gao, Xiuming Jin, Yaying Wu.
